# Candida auris Phenotypic Heterogeneity Determines Pathogenicity *In Vitro*

**DOI:** 10.1128/mSphere.00371-20

**Published:** 2020-06-24

**Authors:** Jason L. Brown, Chris Delaney, Bryn Short, Mark C. Butcher, Emily McKloud, Craig Williams, Ryan Kean, Gordon Ramage

**Affiliations:** a Oral Sciences Research Group, Glasgow Dental School, School of Medicine, College of Medical, Veterinary and Life Sciences, University of Glasgow, Glasgow, United Kingdom; b Department of Biological and Biomedical Sciences, School of Health and Life Sciences, Glasgow Caledonian University, Glasgow, United Kingdom; c Microbiology Department, Lancaster Royal Infirmary, University of Lancaster, Lancaster, United Kingdom; d Glasgow Biofilm Research Network, Glasgow Dental School, Glasgow, United Kingdom; University of Georgia

**Keywords:** *Candida auris*, aggregate, host-pathogen interactions, *in vitro* skin model, heterogeneity

## Abstract

Candida auris is an enigmatic yeast that provides substantial global risk in health care facilities and intensive care units. A unique phenotype exhibited by certain isolates of C. auris is their ability to form small clusters of cells known as aggregates, which have been to a limited extent described in the context of pathogenic traits. In this study, we screened several nonaggregative and aggregative C. auris isolates for biofilm formation, where we observed a level of heterogeneity among the different phenotypes. Next, we utilized an RNA sequencing approach to investigate the transcriptional responses during biofilm formation of a nonaggregative and aggregative isolate of the initial pool. Observations from these analyses indicate unique transcriptional profiles in the two isolates, with several genes identified relating to proteins involved in adhesion and invasion of the host in other fungal species. From these findings, we investigated for the first time the fungal recognition and inflammatory responses of a three-dimensional skin epithelial model to these isolates. In these models, a wound was induced to mimic a portal of entry for C. auris. We show that both phenotypes elicited minimal response in the model minus induction of the wound, yet in the wounded tissue, both phenotypes induced a greater response, with the aggregative isolate more proinflammatory. This capacity of aggregative C. auris biofilms to generate such responses in the wounded skin highlights how this opportunistic yeast is a high risk within the intensive care environment where susceptible patients have multiple indwelling lines.

**IMPORTANCE** Candida auris has recently emerged as an important cause of concern within health care environments due to its ability to persist and tolerate commonly used antiseptics and disinfectants, particularly when attached to a surface (biofilms). This yeast is able to colonize and subsequently infect patients, particularly those that are critically ill or immunosuppressed, which may result in death. We have undertaken analysis on two different phenotypic types of this yeast, using molecular and immunological tools to determine whether either of these has a greater ability to cause serious infections. We describe that both isolates exhibit largely different transcriptional profiles during biofilm development. Finally, we show that the inability to form small aggregates (or clusters) of cells has an adverse effect on the organism’s immunostimulatory properties, suggesting that the nonaggregative phenotype may exhibit a certain level of immune evasion.

## Abstract (precis)

Candida auris has recently emerged as an important cause of concern within health care environments due to its ability to persist and tolerate commonly used antiseptics and disinfectants, particularly when attached to a surface (biofilms). This yeast is able to colonize and subsequently infect patients, particularly those that are critically ill or immunosuppressed, which may result in death. We have undertaken analysis on two different phenotypic types of this yeast, using molecular and immunological tools to determine whether either of these has a greater ability to cause serious infections. We describe that both isolates exhibit largely different transcriptional profiles during biofilm development. Finally, we show that the inability to form small aggregates (or clusters) of cells has an adverse effect on the organism’s immunostimulatory properties, suggesting that the nonaggregative phenotype may exhibit a certain level of immune evasion.

## INTRODUCTION

Candida auris is a nosocomial pathogen first identified in 2009 (1). To date, this multidrug-resistant organism has been identified in more than 40 countries on six different continents, providing a substantial global risk in health care facilities and intensive care units (2–4). It is postulated that the emergence of C. auris may have coincided with climate change based on its particular attributes, resulting in a thermotolerant organism with the ability to persist in the environment before transmission to humans (5).

A unique pathogenic trait exhibited by some isolates of C. auris is their ability to form aggregates (Agg) (6–8). Despite the well-documented prevalence of C. auris worldwide, relatively little is known about the Agg phenotype of the organism. The existence of four geographically and phylogenetically distinct clades of the organism (2), and a fifth recently proposed clade (9), has restricted definitive profiling of the C. auris pathogenic mechanism of these aggregates in regard to biofilm-forming capabilities, drug resistance pathways, and interactions with the host. Of the publications that exist, some have documented characteristic pathogenic traits for both phenotypes *in vitro* and *in vivo* (6, 10, 11). Others have shown that the Agg phenotype is inducible under certain conditions (7, 8), while histological analyses of murine models have shown that aggregates can accumulate in organs following C. auris infection (7, 12, 13). Therefore, further studies are required to investigate this characteristic Agg phenomenon in C. auris isolates to fully comprehend the pathogenic pathways of the organism and to understand how such mechanisms may differ from their nonaggregative (non-Agg) counterparts.

Limited evidence also exists for studies investigating the interactions of C. auris with components of the host, although several *in vivo* models have been employed to document the virulence of C. auris. Of these models, Galleria mellonella larva infection models and murine models of invasive candidiasis have shown various survival rates postinfection with C. auris (6, 8, 10, 12–15), reaffirming that genetic variability among clades impacts the organism’s virulence. However, such studies have been limited in investigating the host immune response to the organism. Recently, Johnson et al. utilized a zebrafish (Danio rerio) model to monitor C. auris-host cell interactions *in vivo* (16). This work highlighted that C. auris (strain B11203 Indian isolate phylogenetically part of the South Asian or India/Pakistan clade (2), which appeared to exhibit a non-Agg phenotype) was resistant to neutrophil-mediated killing, suggesting that the organism has the ability to persist incognito in the host (16). *In vitro* studies have shown interactions between C. auris and epithelial tissue, emphasizing that the organism can persist on skin. In this study, Horton and colleagues demonstrated that C. auris (B11203 strain, as above) formed high-burden biofilms on porcine skin biopsy specimens in the presence of an artificial sweat medium (17). However, to date, no studies have documented the host inflammatory response to the non-Agg and/or Agg phenotype.

In this study, we sought to investigate the level of heterogeneity among different non-Agg and Agg isolates. We deemed this pertinent given that such traits of heterogeneity among isolates have previously been described for other *Candida* species, significantly impacting clinical outcomes and mortality rates (18). To further investigate this Agg versus non-Agg phenotype, transcriptome analyses were performed on planktonic cells and biofilms of two selected isolates from the initial pool. Upon completion of these analyses, we discovered that several genes associated with cell membrane and/or cell wall proteins (e.g., cellular components) were upregulated in the Agg biofilm. Such unique transcriptional profiles in respect to the cellular components led us to investigate the host response following stimulation with both C. auris phenotypes *in vitro*. For this, a two- and three-dimensional skin wound model was employed to investigate the epithelial response to the Agg and non-Agg isolates of C. auris. Both skin wound models exhibited different profiles to each isolate, indicating unique fungal recognition and/or host response to the Agg and non-Agg phenotype. Interestingly, there was minimal response by the host to C. auris without induction of the wound, suggesting that the organism relies on the loss of tissue integrity to become invasive.

## RESULTS

**FIG 1:**
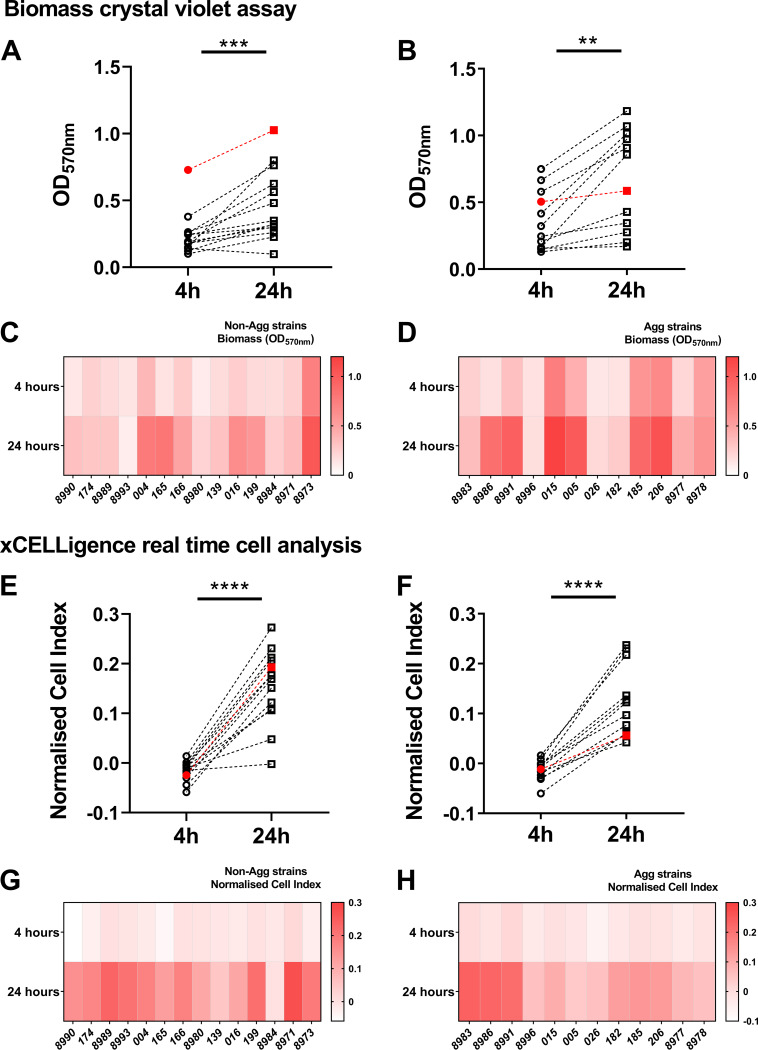
Nonaggregative and aggregative Candida auris biofilm heterogeneity. Biomass and impedance measurements were used as measures of biofilm formation of 26 isolates of C. auris (*n* = 14 for the nonaggregative phenotype and *n* = 12 for the aggregative phenotype). For biomass assessment, 1 × 10^6^ fungal cells were seeded in 96-well plates, and biofilm developed for 4 h or 24 h prior to crystal violet staining. Panels A and B show the differences in absorbance at 570 nm of the nonaggregative and aggregative phenotypes, respectively, at 4 h and 24 h. (C and D) The heatmaps show the average absorbance at 570 nm for each individual isolate at both time points. The formation of C. auris biofilms in real time was monitored using electron impedance measurements on the xCELLigence real-time cell analyzer. (E and F) Electron impedance measurements are presented as cell index for all isolates and for biofilms developed for 4 h and 24 h, respectively. (G and H) The heatmaps depict the mean cell index values for all nonaggregative (G) and aggregative (H) isolates. Red data points indicate the two isolates selected for further analyses in this study (NCPF 8973 and NCPF 8978). Paired Student’s *t* tests were used for statistical analyses, and statistically significant differences for data were determined and are indicated by bars and asterisks as follows: **, *P* < 0.01; ***, *P* < 0.001; ****, *P* < 0.0001.

The Agg phenotype is a unique trait of C. auris, one that can influence the organism’s pathogenic traits *in vitro* and *in vivo* (6, 10, 11). To corroborate these previous observations, differences in early and late biofilm formation were assessed between non-Agg and Agg isolates of C. auris. A total of 26 non-Agg and Agg C. auris clinical isolates were screened during early (4-h) and mature (24-h) biofilm growth stages (Fig. 1A and B). Both sets of non-Agg and Agg C. auris isolates formed biofilms in a time-dependent manner as assessed by crystal violet staining. With the exception of C. auris NCPF 8993 (non-Agg) and NCPF 8996 (Agg), all isolates formed biofilms with greater biomass after 24-h culture than after 4 h of culture (Fig. 1C and D). Although the Agg phenotype tended to form biofilms with greater biomass at both 4 h and 24 h, we observed no statistical differences when collectively comparing all nonaggregating and aggregating isolates of C. auris at either time point (see Fig. S1A and B in the supplemental material). From these data, it was clear that some isolates formed biofilms with greater biomass than others, suggestive of a certain level of heterogeneity between isolates of Agg and non-Agg phenotype, in line with previous observations (6, 10, 19). Similar trends of biofilm heterogeneity were observed when monitoring biofilm formation via impedance measurements in real time at both time points in non-Agg and Agg phenotype (Fig. 1E to H and Fig. S1C and D). These data support corroboration of these unique phenotypes among isolates.

10.1128/mSphere.00371-20.1 FIG S1 Candida auris heterogeneity between isolates. Biomass and impedance measurements for 26 isolates of C. auris (*n* = 14 for the nonaggregative phenotype and *n* = 12 for the aggregative phenotype) are shown as described in the legend to Fig. 1. Panels A and B depict the biomass of the isolates determined using crystal violet staining of 4-h (A) and 24-h (B) biofilms. Electron impedance measurements are presented as cell index values for all isolates, for biofilms developed for 4 h (C) and 24 h (D). Red data points indicate the two isolates selected for further analyses in this study (NCPF 8973 and NCPF 8978). No statistical significance was observed in the data (*P* > 0.05). Download FIG S1, DOCX file, 0.9 MB. Copyright © 2020 Brown et al. 2020 Brown et al. This content is distributed under the terms of the Creative Commons Attribution 4.0 International license.

**FIG 2:**
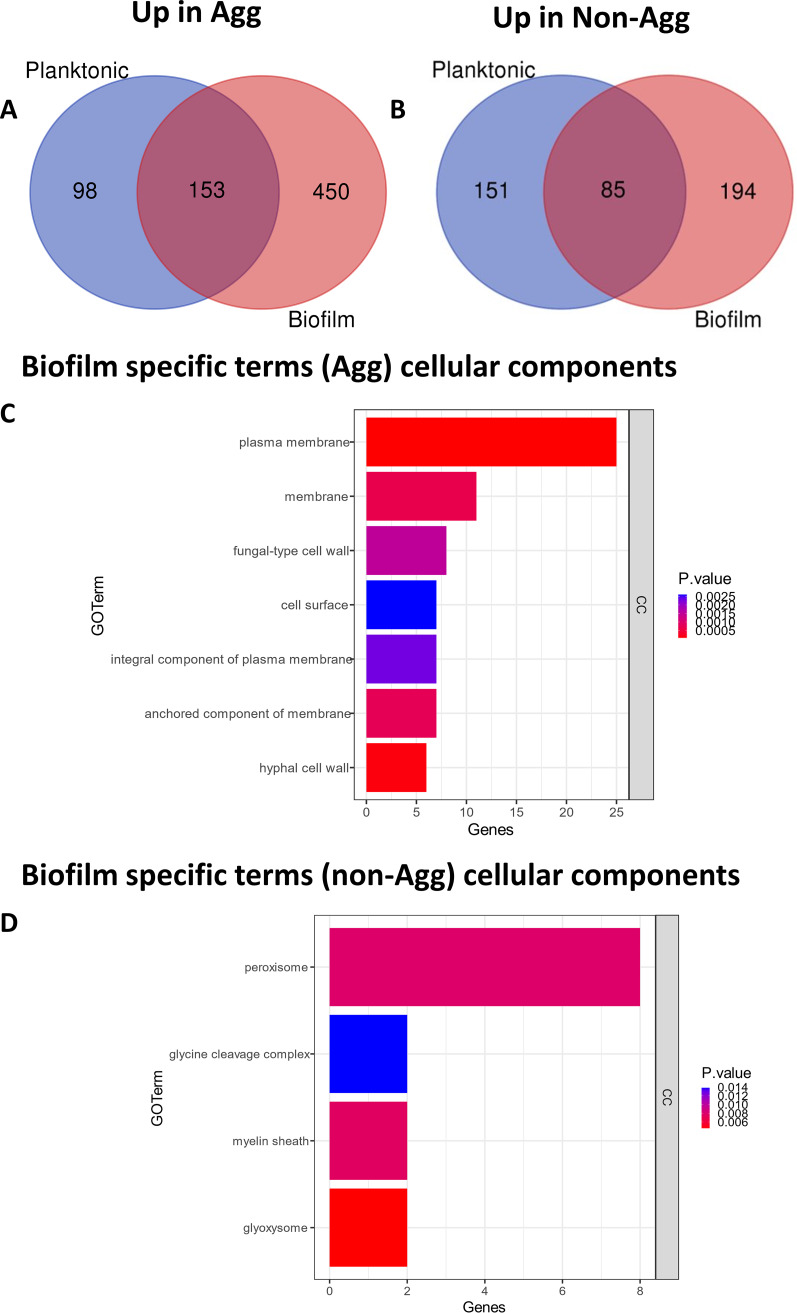
Transcriptional profile of nonaggregative and aggregative Candida auris during biofilm formation. RNA from planktonic cells and biofilms formed for 24 h of two isolates (NCPF 8973 and NCPF 8978) was used for RNA sequencing and transcriptome analyses as described in the text. (A and B) Venn diagrams depict upregulated genes in aggregative (A) or nonaggregative (B) phenotype in either the planktonic form (blue circle) or the biofilm form (red circle) or in both forms (blue and red circle). (C and D) Gene distribution of significantly upregulated genes in biofilm forms were grouped for gene ontology analysis. Genes upregulated that belong to the functional pathway cellular components (CC) are shown, while the other two pathways (biological processes [BP] and metabolic functions [MF], respectively) are included in Fig. S2 in the supplemental material. A cutoff twofold upregulation was used for gene ontology analysis using an adjusted *P* value of <0.05.

To further study the pathogenic and biofilm-forming characteristics of Agg and non-Agg C. auris, transcriptional profiling of 24-h planktonic versus biofilm phenotypes was performed. For these studies, two clinical isolates from the initial pool tested were selected for analysis (non-Agg NCPF 8973 and Agg NCPF 8978, indicated by the red points in Fig. 1). First, we found that a total of 701 genes were upregulated in planktonic and/or biofilm form of Agg compared to the non-Agg phenotype, of which 450 genes were upregulated in the biofilm state (Fig. 2A). Conversely, fewer genes (430) were upregulated in non-Agg C. auris in the planktonic, biofilm, or both states compared to Agg C. auris counterparts, with 194 genes upregulated in the biofilm form (Fig. 2B). In order to understand the functional processes related to differentially expressed genes, a cutoff of twofold upregulation was used for gene ontology (GO) analysis (adjusted *P* value of <0.05). Upregulated genes in non-Agg versus Agg C. auris biofilms normalized to planktonic cell expression involved three functional classes: biological processes (BP), cellular components (CC), and metabolic functions (MF) (Fig. S2A and B). Interestingly, most genes upregulated in the Agg biofilm belonged to the CC functional class; more than 40 genes related to membrane and cell wall constituents were upregulated in the Agg form compared to the non-Agg phenotype (Fig. 2C). Several genes associated with fungal cell wall proteins were upregulated in the Agg biofilms, including *TSA1*, *ECM33*, *MP65*, and *PHR1* (Fig. S2C). Moreover, included in these 40 genes were members of the *ALS* family of adhesins such as *ALS1*. In contrast, in the non-Agg biofilms, the greatest changes in expression were observed for genes belonging to functional classes of BP and MF (Fig. S2C). Only a small number of genes belonging to cellular components were upregulated in the non-Agg biofilm compared to the Agg biofilm (Fig. 2D). Of these genes, <20 genes belonging to the peroxisome, glycine cleavage complex, myelin sheath, and glyoxysome were upregulated in the non-Agg isolate.

10.1128/mSphere.00371-20.2 FIG S2 Transcriptional profiles of aggregative and nonaggregative Candida auris. Functional annotation of upregulated C. auris genes in 24-h biofilms of NCPF 8978 (A) and NCPF 8973 (B) isolates are shown, grouped into biological process (BP), cellular component (CC), and metabolic function (MF) gene ontology categories. Log_2_ fold change of genes associated with cell wall components upregulated in aggregative biofilms are shown in panel C. A cutoff of twofold upregulation was used for gene ontology analysis using an adjusted *P* value of <0.05. Download FIG S2, DOCX file, 0.9 MB. Copyright © 2020 Brown et al. 2020 Brown et al. This content is distributed under the terms of the Creative Commons Attribution 4.0 International license.

**FIG 3:**
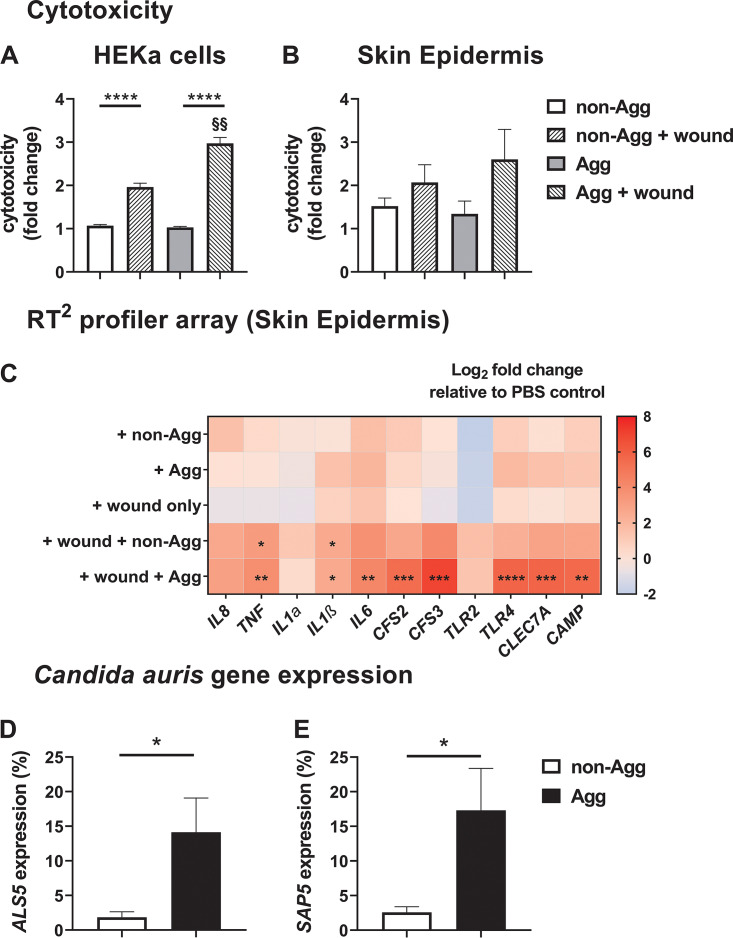
Cytotoxic and inflammatory effects of aggregative and nonaggregative Candida auris on skin epithelial models *in vitro*. For these analyses, a two- and three-dimensional skin epithelial model was used as schematically shown in Fig. 4. (A and B) First, cytotoxicity in the models was determined by quantifying the amount of lactate dehydrogenase (LDH) released by the human adult epidermal keratinocytes (HEKa) (A) and skin epidermis (B) following coculture with the aggregative (NCPF 8978) and nonaggregative (NCPF 8973) isolates of C. auris. For this, data were presented as fold change relative to the value for the PBS control. (C) To study the host response to C. auris, an RT^2^ profiler array containing genes associated with inflammation and fungal recognition was utilized to assess the transcriptional profile of the skin epidermis following stimulation. Data in the heatmap are presented as log_2_ fold change relative to the value for the PBS control. (D and E) Finally, expression of two virulence genes, *ALS5* and *SAP5*, was determined in the isolates. Values are presented as percent expression relative to the fungus-specific housekeeping gene, *β-actin*. All epithelial cells or tissues were infected in triplicate, and statistical significance was determined from raw data threshold cycle (*C_T_*) values using unpaired Student’s *t* tests for comparison of two variables or one-way ANOVA with Tukey’s multiple-comparison posttest for more than two variables (*, *P* < 0.05; ** and §§, *P* < 0.01; ***, *P* < 0.001; ****, *P* < 0.0001).

The observed differences in the transcriptional profiles of genes belonging to the CC of C. auris could impact the ability of the host to recognize the two phenotypes. For example, key cellular components such as ALS proteins of other *Candida* species, such as Candida albicans play important roles in aiding host colonization and orchestrating the innate immune response (20, 21). However, it is currently unknown whether similar mechanisms exist for C. auris. Therefore, the following section investigates whether a non-Agg or Agg phenotype dictates the response by the host to C. auris. For this, a two- and three-dimensional skin epithelial model was employed to study the host response to the two C. auris isolates used above. For both coculture skin systems, a wound was induced to mimic the possible entry site of patients for C. auris in health care environments. In the two-dimensional model containing adult human epidermal keratinocytes (HEKa) cells, both isolates were significantly more cytotoxic to host cells following induction of the wound (*P* < 0.001) (Fig. 3A). Intriguingly, Agg C. auris was significantly more cytotoxic than the non-Agg form in the wound model (*P* < 0.01) (Fig. 3A) It is noteworthy that wounded monolayers minus inoculum were comparable to untreated monolayers (data not shown), suggesting that induction of the wound did not induce cytotoxic effects on the cells. A similar trend in cytotoxicity was observed between the two isolates in the three-dimensional model (Episkin, SkinEthic reconstructed human epidermis [RHE]), although this did not reach statistical significance (Fig. 3B). Interestingly, cytotoxicity of Agg and non-Agg C. auris were comparable in both coculture models minus wounds.

To further study the host response in the three-dimensional system, a transcriptional response in the tissue was investigated using a RT^2^ profiler array containing primers specific for genes associated with inflammatory responses and/or fungal recognition (Fig. 3C). Upon investigation, it was evident that the greatest changes in gene expression were observed in the wound models for both C. auris isolates. Importantly, induction of the wound minus C. auris did not significantly alter the expression of any of the genes arrayed. In the wound model, proinflammatory cytokine genes *TNF* and *IL1β* (*P* < 0.05) were significantly upregulated in RHE tissue cultured with non-Agg C. auris (Fig. 3C). Conversely, the Agg phenotype of C. auris induced the greatest changes in RHE; the expression of 8 of the 11 genes profiled (*IL1β*, *P* < 0.05; *TNF*, *IL-6*, *CAMP*, *P* < 0.01; *CFS2*, *CFS3*, *CLEC7A*, *P* < 0.001; *TLR4*, *P* < 0.0001) (Fig. 3C) were all significantly upregulated following coculture compared to the untreated tissue.

In the three-dimensional coculture system, it was evident from histological and fungus-specific periodic acid-Schiff (PAS) staining that both isolates of C. auris had adhered to the peripheral keratinized layer of the RHE tissue (Fig. S3). Uninfected tissue displayed a well-organized multilayered structure characteristic of skin epidermis *in vivo* (22) (Fig. S3A). However, in infected tissue, there was no sign of C. auris infiltration into the tissue (Fig. S3A and B) as seen with previous publications of skin tissue infection models with invasive C. albicans (23, 24). Unfortunately, loss of tissue integrity in the wounded model rendered them unsuitable for histological staining; therefore, at this juncture, we were unable to visualize whether C. auris invaded the tissue at the wound site. Nonetheless, given that the expression of several important cell membrane and cell wall proteins are differentially regulated in non-Agg and Agg phenotypes of C. auris biofilms, we wanted to assess the gene response of C. auris in the infected tissue. For this, two key virulence factor genes belonging to the *ALS* and *SAP* families were selected for analyses. Both these gene families are involved in translation of two key proteins associated with *Candida* invasion of the host and associated virulence (25–27). The two genes selected, *ALS5* and *SAP5*, were both upregulated in the Agg phenotype compared to the non-Agg C. auris isolate in the three-dimensional tissue model (Fig. 3D and E). Such a response in the Agg isolate may begin to elucidate the mechanism by which this phenotype generated a greater inflammatory response within the tissue.

10.1128/mSphere.00371-20.3 FIG S3 Histological features of the three-dimensional skin epidermis with and without Candida auris infection. Reconstructed human epidermis (RHE) was inoculated with PBS only (A), nonaggregative (non-Agg) C. auris NCPF 8973 (B), or aggregative (Agg) C. auris NCPF 8978 (C) for 24 h at 37°C and 5% CO_2_. Following incubation, tissues were fixed in 10% formalin and then processed for histology. Haematoxylin-and-eosin stain was used to assess the histological properties of the skin epidermis, showing that the tissue formed a multilayered structure with keratinized peripheral cell layer (left panels). The fungus-specific periodic acid-Schiff (PAS) reagents were used to identify the fungal cells in the tissue. These cells were counterstained with hematoxylin (right panels). The PAS staining depicts attachment of the fungal cells to the keratin layer, as indicated by the black arrows (B and C). Download FIG S3, DOCX file, 0.6 MB. Copyright © 2020 Brown et al. 2020 Brown et al. This content is distributed under the terms of the Creative Commons Attribution 4.0 International license.

## DISCUSSION

Results of this study further indicate that the Agg phenotype of C. auris determines its pathogenicity *in vitro*. This phenotype, first reported by Borman et al., is characterized in certain isolates by the formation of individual yeast cells mixed with large aggregations in planktonic form (6). This aggregating behavior was later shown to affect biofilm formation, antifungal susceptibility, and virulence of the organism (6, 10, 11, 19). Moreover, aggregation has recently been found to be an inducible trait triggered by subinhibitory concentrations of triazole and echinocandin antifungals, suggesting that treatment regimens must be carefully considered to combat C. auris dependent on its Agg phenotype (8). In this study, we report a previously described RNA-sequencing approach (28) in order to compare the transcriptional responses of one non-Agg isolate (NCPF 8973) and one Agg isolate (NPCF 8978) of C. auris during formation of biofilms from planktonic cells. These analyses indicated that several key cell membrane and cell wall components were upregulated in Agg biofilms, many of which are involved in cell adhesion to abiotic surfaces and host cells. These observations led us to pose the question: how would the host respond to the two phenotypes? As such, we document for the first time an investigation into the host skin epithelial response to Agg and non-Agg C. auris.

Given the clear differences in virulence traits of the non-Agg and Agg phenotype, we deemed it pertinent to study in greater detail the transcriptional profiles of one non-Agg isolate and one Agg isolate of C. auris. These two isolates displayed the heterogeneity exhibited by other Agg and non-Agg isolates, with the NCPF 8973 isolate forming a denser biofilm after 24 h (as shown here in Fig. 1 by the red data points and elsewhere [10]). It must be noted here that the non-Agg C. auris isolates showed high variation in biofilm-forming capabilities, with the NCPF 8973 isolate forming a biofilm with greater biomass than the other isolates in the group. In contrast, the Agg isolate selected (NCPF 8978) was an intermediate biofilm former among its group. Nevertheless, irrespective of the biofilm-forming capabilities, transcriptome analyses showed that an increased number of genes was upregulated in the Agg biofilm compared to the non-Agg biofilm of the two isolates selected (450 versus 194 genes, respectively; Fig. 2), suggestive that the clustering of aggregated cells greatly impacts the transcriptome of the organism during the formation of a biofilm. Of these differential responses in the two biofilm phenotypes, a vast number of genes upregulated in the Agg biofilm belonged to CC as assessed using GO analyses. Specifically, several cell wall genes were upregulated in the Agg phenotype, e.g., *TSA1*, *ECM33*, *MP65*, *ALS1*, and *PHR1*. In C. albicans, ECM33 and MP65 have been deemed important proteins in maintenance of fungal cell wall integrity, biofilm formation, and stress responses (29–31). Furthermore, the ALS family of adhesin proteins and PHR family of extracellular transglycosylases also function as important regulators of biofilm formation in C. albicans (32, 33). For example, loss of function of PHR and ALS proteins results in impaired adhesion and biofilm development (34, 35). It must be noted here that such direct comparisons in transcriptional responses between findings on C. auris and C. albicans biofilms must be taken with a certain degree of caution, given the lack of true hyphal formation by C. auris isolates grown in biofilms (6), although such a trait can be induced under some conditions (7, 14). An absence of hyphae in C. auris during biofilm formation could impact the transcriptional response by the organism compared to other *Candida* species, especially given that proteins such as ALS have been shown to be upregulated and involved in the yeast-to-hypha transition in C. albicans (36, 37). Nevertheless, such in-depth analyses, as those documented herein, may begin to explain the mechanisms behind aggregate formation in biofilms of some C. auris isolates.

Most of the aforementioned genes (*TSA1*, *ECM33*, *MP65*, *ALS1*, and *PHR1*) have multiple functions in C. albicans pathogenicity, particularly in biofilm formation as discussed above. In addition, most also play key roles in attachment and/or survival within the host in other fungal species. For example, *TSA1*, which encodes a protein called thiol-specific antioxidant 1, has been identified in C. albicans and Cryptococcus neoformans, and functions as an important stress response regulator in unfavorable oxidative environments (38, 39), potentially those generated by the host (40). In C. albicans, *ECM33*, *MP65*, and *PHR1* have been shown to be important genes necessary for production of proteins involved in adherence and invasion of host cells (29–31, 34). For example, in similar three-dimensional reconstituted skin and oral epithelial coculture models, heterozygous mutants of *ECM33* and *PHR1* displayed clear deficiencies in penetration of epithelial cell layers leading to reduced tissue invasion and subsequent cellular damage (30, 34). Finally, another gene upregulated in Agg C. auris biofilms was *ALS1*, a member of the ALS family of adhesin proteins. This cohort of adhesin proteins which contains at least eight members in C. albicans are well-documented virulence factors, particularly in host-pathogen interactions *in vitro* and *in vivo* (25, 32, 41). Concerning the results shown in this study, the role of *ALS1*, which encodes the protein Als1p, in *Candida*-host interactions remains unclear. As such, contradictory reports state a role for this adhesion in attachment to epithelial cells. Kamai and colleagues showed that heterozygous knockouts of *ALS1* were unable to colonize oral tissues of mice *in vivo* and *ex vivo* as efficiently as wild-type strains or knockouts coupled with the Als1p reinstated (42). In contrast, it was shown that attachment of the C. albicans *ALS1* null mutant to oral epithelial cells was no different than that of wild-type controls, suggesting that its role in adhesion was not as important as other *ALS* family members (26). Nevertheless, as discussed briefly above, such comparisons between C. auris and C. albicans interactions with host cells must be tentatively compared given the differences in the morphological forms of the two species. In particular, the transition from yeast to hyphae in C. albicans is essential for tissue invasion of mucosal surfaces. For example, fungal invasion mechanisms have been identified that show morphological changes from yeast to pseudohyphae to hyphae during the process of adhesion and invasion in C. albicans in epithelial tissue (43, 44). This process is even essential for the discrimination of commensal and pathogenic forms of C. albicans by the host (45). Given its inability to form hyphae under normal physiological conditions, how C. auris can invade epithelial tissue is unknown. It is possible that the organism has different invasive mechanisms from other *Candida* species, including possible variations among isolates from different clades. However, at this juncture, further studies are necessary to elucidate such mechanisms, including consideration of the virulence properties of various non-Agg and Agg isolates.

It has been postulated that C. auris exhibits a level of immune evasion to bypass immunological defenses (4). Recent work by Johnson et al. showed that C. auris was resistant to neutrophil-mediated killing by failing to stimulate neutrophil elastase trap (NET) formation both *in vitro* and *in vivo* in a zebrafish infection model (16). Another study found that viable C. auris did not induce an inflammatory response in human peripheral blood mononuclear cells (PBMCs), although fungal cells were recognized and engulfed by human monocyte-derived macrophages. Conversely, such host responses were significantly greater against other *Candida* species such as Candida tropicalis, Candida guilliermondii, and Candida krusei (46). It is apparent from these studies that the host recognizes yet fails to generate an effective immune response against C. auris. From the results described here, it is evident that C. auris is not cytotoxic nor proinflammatory to intact skin epithelial cells or epidermis tissue. Only following induction of a wound in these models did C. auris elicit any significant response by the host. From this, it could be suggested that under normal immunological conditions, the organism is noninvasive, and any immune response from the host is minimal. Confirming this, previous studies have shown that immunocompetent mice were more resistant to C. auris infection than immunocompromised mice (15). Such findings have been seen in humans; invasive C. auris infections generally occur in critically ill patients with serious underlying medical conditions resulting in hematological deficiencies and/or immunosuppression (47–49). These observations from animal models and human studies are suggestive that an effective immune system is required to prevent C. auris infection. In the context of this study, the observed noninvasive phenotype of C. auris may simply be due to lack of hyphal formation, whereby yeast cells colonize the periphery of the skin yet do not invade the underlying layers unless in the presence of a wound. Indeed, similar observations have been made elsewhere. Horton et al. recently showed that C. auris forms layers of cells on the periphery of porcine skin *ex vivo*, but these C. auris structures were devoid of pseudohyphae or hyphae (17). Until further studies commence, we cannot comment on whether the observed interactions with the host tissue are homogenous among the non-Agg and Agg phenotypes.

A limitation from previous studies is the failure to investigate the host response to the Agg phenotype expressed by certain isolates. We and others have previously shown that the non-Agg isolates of C. auris are more virulent in G. mellonella larval models than the Agg counterparts, possibly resulting from enhanced dissemination rates of the single cells (6, 10). Here, the Agg NCPF 8978 isolate was significantly more cytotoxic and proinflammatory than non-Agg NCPF 8973 in the two- and three-dimensional skin models. At this juncture, it is unknown why such a response occurs in the host. Murine models of candidiasis have shown that C. auris can accumulate in the kidneys of mice in the form of aggregates (7, 12, 13), suggesting that aggregation may occur *in vivo* to enable persistence and survival. However, to date, no studies have investigated the host response to such C. auris aggregates *in vivo*. It could simply be that the Agg phenotype generates a cluster of cells with increased pathogenic traits to induce a greater host response than single cells. This was confirmed by the upregulation of two key adhesin and proteinase genes, *ALS5* and *SAP5*, in the Agg NCPF 8978 isolate compared to non-Agg NCPF 8973 in the skin epidermis model. Interestingly, similar observations have been reported for biofilm-dispersed single cells versus aggregates in model bacteria such as Pseudomonas aeruginosa. As such, dispersed aggregates of P. aeruginosa possess enhanced antibiotic resistance traits, likely due to encapsulation by extracellular matrix, and greater immune evasion techniques over dispersed single cells (50–52). Conversely, it could be argued that the single-cell phenotype of C. auris exhibits a level of immune evasion (as postulated elsewhere [16, 53]), which may explain the lack of response by the host to this phenotype. Future studies must continue to investigate the unique Agg phenotype of C. auris to fully clarify the organisms’ pathogenic mechanisms. These investigations must consider interactions between C. auris and other organisms that comprise the skin and/or wound microbiomes, which may function as important beacons for host invasion of C. auris.

The identification of five geographically and phylogenetically distinct clades of C. auris (2, 9), containing isolates capable of forming aggregates with enhanced drug resistance, has meant that unravelling the pathogenic mechanisms employed by C. auris *in vitro* and *in vivo* remains extremely difficult. This study has highlighted different pathogenic signatures of Agg and non-Agg forms of C. auris in biofilms and during host invasion *in vitro*, albeit with only one other isolate from each phenotypic group. Of course, given the level of heterogeneity among isolates, in-depth analyses from this work are limited to one Agg isolate and one-Agg isolate. At this juncture, it is unknown whether other isolates behave in a manner similar to the two isolates described in this study, and this warrants further consideration moving forward. The level of heterogeneity observed among isolates in regard to the non-Agg/Agg phenotype may arise from different unique transcriptional responses between the groups. As such, future studies must continue to investigate these unique phenotypic traits of different Agg and non-Agg isolates of C. auris from different clades to fully understand the persistence of this nosocomial pathogen in the health care environment and whether such traits are comparable among the diverse isolates.

## MATERIALS AND METHODS

### Microbial growth and standardization.

For *in vitro* biofilm biomass assessment, a pool of aggregating (Agg; *n* = 12) and single-celled, nonaggregative (non-Agg; *n* = 14) C. auris clinical isolates (gift from Andrew Borman and Elizabeth Johnson, Public Health England, UK). The isolates used and the clades they belong to are shown in Table 1. All C. auris isolates were stored in Microbank beads (Pro-Lab Diagnostics, UK) prior to use. Each isolate was grown on Sabouraud dextrose (SAB) agar (Oxoid, UK) at 30°C for 24 to 48 h and then stored at 4°C prior to propagation in yeast-peptone-dextrose (YPD; Sigma-Aldrich, UK) medium overnight (16 h) at 30°C, gently shaking at 200 rpm. The cells were pelleted by centrifugation (3,000 × *g*) and then washed two times in phosphate-buffered saline (PBS). The cells were then standardized to the desired concentration after counting using a hemocytometer and then resuspended in selected media for each assay, as described in this article.

**TABLE 1:** Candida auris isolates used in this study^*a*^

Agg phenotype	Isolate	Clade
Non-Agg	8990	Indian
	174	Indian
	8989	Indian
	8993	Indian
	13004	Indian
	165	Indian
	166	Indian
	8980	African
	139	African
	13016	African
	199	African
	8984	Japan/Korea
	8971	Indian
	8973*	Indian

Agg	8983	Indian
	8986	Indian
	8991	Indian
	8996	African
	13015	African
	13005	African
	13026	African
	182	African
	185	African
	206	African
	8977	African
	8978*	African

a All isolates used in the preliminary experiments in this study (as seen in Fig. 4). The aggregative phenotype and clade for each isolate are also shown. The two isolates (NCPF 8973 and NCPF 8978) selected for transcriptional analyses and coculture experiments are indicated by an asterisk.

C. auris isolate phenotypes were determined visually by suspending one colony in 1 ml of PBS (Sigma-Aldrich, UK). Isolates were termed “aggregators” if the added colony did not disperse upon mixing in PBS. For RNA sequencing and transcriptional analysis of C. auris biofilms and coculture systems, one Agg isolate (NCPF 8978) and one non-Agg isolate (NCPF 8973) were used.

### Biofilm growth and biomass assessment.

Fungal cells were adjusted to 1 × 10^6^ cells/ml in Roswell Parks Memorial Institute (RPMI) medium (Sigma-Aldrich, UK), and biofilms formed for 4 or 24 h at 37°C in flat-bottom wells of 96-well plates (Corning, UK). Appropriate medium controls were included on each plate to test for contamination. Following incubation, biofilms were washed gently once in PBS to remove any nonadhered cells. The biomass of each biofilm was determined via 0.05% crystal violet (CV) staining as described previously (54). Absorbance of the CV stain was measured spectrophotometrically at 570 nm in a microtiter plate reader (FLUOStar Omega, BMG Labtech, UK).

### Monitoring the growth of Candida auris biofilms in real time.

The xCELLigence real-time cell analyzer (RTCA; ACEA Bioscience Inc., San Diego, CA) was used to monitor the formation of C. auris biofilms in real time using electron impedance measurements (presented as cell index [CI]) which is directly related to cell attachment and proliferation. In brief, the E-plate containing 100 μl of preheated RPMI medium was loaded into the RTCA which had been placed in the incubator 2 h prior to the experiment to test medium impedance and electrode connectivity. The cultures of each C. auris isolate used in this study were standardized to 2 × 10^6^ CFU/ml and added to the E-plate in 100-μl aliquots in triplicate. Appropriate medium controls minus inoculum were also included in triplicate. Biofilm formation was measured over 24 h with CI readings taken every 5 min. Normalized CI values were exported from the RTCA software and analyzed in GraphPad Prism (version 8; GraphPad Software Inc., La Jolla, CA). More-detailed descriptions of this technology can be found elsewhere (55).

### Two-dimensional monolayer coculture model.

Adult human epidermal keratinocyte (HEKa) cells (Invitrogen, Gibco, UK) were used for two-dimensional coculture experiments. Frozen stocks of HEKa cells (1 × 10^6^ cells/ml; passage number lower than 10) were revived and seeded in T-75 tissue culture flasks (Corning, UK) in medium 154 (Thermo-Fisher, UK) supplemented with 100 U/ml of penicillin (Pen)/streptomycin (Strep) and human keratinocyte growth supplement (HKGS) (Thermo-Fisher, UK). The flasks were incubated at 37°C (5% CO_2_), and the medium was changed every 48 h until the cells reached 80 to 90% confluence (56). Confluent cells were passaged using 0.05% trypsin EDTA (Sigma-Aldrich, UK), and the enzymatic reaction was inhibited using trypsin neutralizer solution (Sigma-Aldrich, UK). Passaged cells were then seeded into 24-well plates (Corning, UK) at a final concentration of 2 × 10^5^ cells/ml. After 24 to 48 h, the cells reached adequate confluence for C. auris coculture experiments as described below.

### Three-dimensional human epidermis coculture model.

Reconstituted human epidermis (RHE) used for 3D coculture experiments was purchased from Episkin (Skin Ethic; Episkin, Lyon, France). RHE was formed from healthy human keratinocytes cultured on an inert polycarbonate filter at the air-liquid interface, in a chemically defined medium grown to 17-day maturity. This model is histologically similar to *in vivo* human epidermis. Upon arrival and prior to experimental set-up, RHE was incubated with maintenance medium in 24-well plates (Corning, UK) for 24 h with 5% CO_2_ at 37°C. Maintenance medium was replaced, and then the coculture three-dimensional system was set up as described below.

### Wound model in two-dimensional and three-dimensional coculture systems.

HEKa cell monolayers were scratched using a method similar to a previously described method to mimic a wound model (57, 58). Briefly, monolayers were grown to confluence as described above, and then three parallel scratches were introduced across the surface using a 100-μl pipette tip prior to inoculation with C. auris. For RHE, a 19-gauge needle was used to scratch the tissue. For all coculture experiments, Agg C. auris NCPF 8978 and non-Agg C. auris NCPF 8973 were grown as described above and then standardized to 2 × 10^6^/ml (multiplicity of infection [MOI] of 10 to HEKa cells; MOI of 10 and as previously described for *Candida*-tissue coculture [23]). For the two-dimensional system, 2 × 10^6^/ml C. auris was prepared in 500 μl supplemented medium 154 and added directly to the confluent HEKa cells. For the three-dimensional coculture model, 2 × 10^6^/ml C. auris was prepared in 100 μl of sterile PBS, and this suspension was added directly to the RHE tissue. All experiments were conducted for 24 h at 5% CO_2_. Infected nonscratched HEKa cells and RHE tissue were used as controls, e.g., no wound, and uninoculated cocultured cells or tissues were also included for all experiments. All control and wounded models were infected in triplicate with both isolates of C. auris.

### Histological staining of skin epidermis.

Following coculture with C. auris, epithelial tissue was carefully cut from the 0.5-cm^2^ insert using a 19-gauge needle and washed three times in sterile PBS to remove nonadherent cells in a manner similar to the previously described method (59), as summarized in the schematic in Fig. 4. Tissue was then fixed in 10% neutral buffered formalin prior to embedding in paraffin. A Finnesse ME+ microtome (Thermo Scientific, UK) was used to cut 2-μm sections, and tissue sections were stained with hematoxylin and eosin or with the fungus-specific periodic acid-Schiff (PAS) reagents and counterstained with hematoxylin.

**FIG 4:**
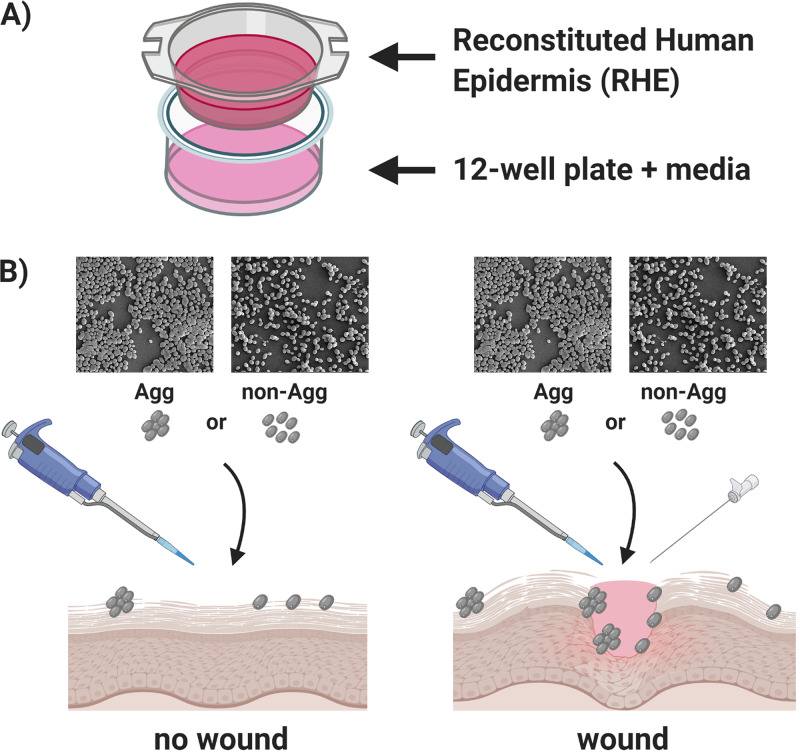
Schematic diagram depicting the experimental set up for the three-dimensional coculture of skin epidermis and Candida auris. A 17-day mature reconstructed human epidermis (RHE) on 0.5-cm^2^ inserts was purchased from Episkin (Skin Ethic). (A) Inserts were carefully lowered into 24-well plates containing maintenance medium supplied by the company. (B) To assess the host response to aggregative and nonaggregative C. auris, control and wounded tissue was cocultured with both isolates (NCPF 8973 and NCPF 8978). A total of 2 × 10^6^ fungal cells in 100 μl PBS was added to the tissue and incubated overnight at 37°C and 5% CO_2_. For some tissues, prior to the addition of fungal inoculum, three scratch wounds were induced using a sterile 19-gauge needle across the surface of the tissue. For visual representation of phenotype, scanning electron microscopy images of 24-h biofilms are included in panel B, clearly showing the differences in cellular phenotypes between the two C. auris isolates. These images were taken at ×1,000 magnification as viewed under a JEOL JSM-6400 scanning electron microscope (samples processed as previously described [70]). Image created using Biorender.

### Epithelial cell viability.

To assess any cytotoxic effects of C. auris on HEKa cells and RHE tissue, a Pierce lactate dehydrogenase (LDH) cytotoxicity assay kit (Thermo Scientific, UK) was used according to the manufacturers’ instructions. Following coculture, cell or tissue spent medium was assayed using the above kit to quantify the level of LDH release as a measure of host cellular disruption.

### Differential gene expression analysis.

HEKa cells and RHE tissue following coculture were lysed in RLT lysis buffer (Qiagen Ltd., UK) containing 0.01% (vol/vol) β-2-mercaptoethanol (β2ME) before bead beating. All RNA was extracted using the RNeasy minikit according to the manufacturer’s instructions (Qiagen Ltd., UK) and quantified using a NanoDrop 1000 spectrophotometer (Thermo Scientific, UK). RNA was converted to complementary DNA (cDNA) using the High Capacity RNA to cDNA kit (Life Technologies, UK) per the manufacturer’s instructions. Gene expression was assessed using SYBR Green^ER^ based-quantitative PCR (qPCR) or RT^2^ profiler arrays (Qiagen Ltd., UK). For SYBR Green^ER^ based-qPCR analyses, the following PCR thermal profiles was used; holding stage at 50°C for 2 min, followed by denaturation stage at 95°C for 10 min, and then 40 cycles, with 1 cycle consisting of 95°C for 3 s and 60°C for 15 s. qPCR plates were run on the StepOnePlus real-time PCR system. The following primer sequences were used for SYBR Green^ER^-based-qPCR analyses of host cells: for *GAPDH*, forward primer, 5′ to 3′, CAAGGCTGAGAACGGGAAG, and reverse primer, 5′ to 3′, GGTGGTGAAGACGCCAGT (60); for *IL-8*, forward primer, 5′ to 3′, CAGAGACAGCAGAGCACACAA, and reverse primer, 5′ to 3′, TTAGCACTCCTTGGCAAAAC (61). For gene expression analyses of C. auris, primers for adhesin gene *ALS5* and proteinase gene *SAP5* were used as follows: for *ALS5*, forward primer, 5′ to 3′, ATACCAGGGTCGGTAGCAGT, and reverse primer, 5′ to 3′, CTATCTTCGCCGCTTGGGAT, and for *SAP5*, forward primer, 5′ to 3′, GGATGCAGCTCTTCCTGGTT, and reverse primer, 5′ to 3′, CTTCCAGTTTGCGGTTGTGG. For other gene expression analyses of RHE tissue, a custom-designed RT^2^ Profiler PCR array was compiled containing primers for genes associated with inflammation and fungal recognition or stimulation of host tissue. For these arrays, the following thermal cycle was used on the MxProP quantitative PCR machine; 10 min at 95°C, followed by 40 cycles, where 1 cycle consisted of 15 s at 95°C and 60 s at 60°C. Data were assembled using MxProP 3000 software (Stratagene, Netherlands). Expression levels for all genes of interest were normalized to the housekeeping gene, *β-actin* for C. auris gene expression and *GAPDH* for mammalian cells, according to the 2^−ΔCt^ method, and then quantified using the 2^−ΔΔCt^ method (62).

### RNA sequencing.

For RNA sequencing of C. auris biofilms, RNA was extracted from 24-h C. auris biofilms as described previously (63). In brief, biofilms were grown as described above on Thermanox coverslips (Thermo-Fisher, UK) in 24-well plates (Corning, UK). The biofilms were removed from the coverslips by sonication at 35 kHz for 10 min in a sonic bath in 1 ml of PBS, and the sonicate was transferred to a 2.0-ml RNase-free bead beating tube (Sigma-Aldrich, UK). The cells were homogenized in TRIzol (Invitrogen, UK) with 0.5-mm glass beads using a BeadBug microtube homogenizer for a total of 90 s (Benchmark-Scientific, USA). RNA was then extracted as described above using the RNeasy minikit according to the manufacturer’s instructions (Qiagen Ltd., UK). Following extraction, RNA quality and quantity were determined using a Bioanalyzer (Agilent, USA), where a minimum RNA integrity number and quantity of 7 and 2.5 μg, respectively, were obtained for each sample. Annotation of data following sample submission to Edinburgh Genomics (http://genomics.ed.ac.uk/) was completed as previously described (28). Briefly, raw fastq reads were trimmed and aligned to the Candida auris representative genome B8441 using Hisat2 (64). Reads were then processed and assembled *de novo* using the Trinity assembly pipeline (65). Trinotate and Blast2Go were utilized to assign gene identifiers (IDs) to homologous sequences using BLAST and Interpro (66, 67). Differential expression analysis was performed according to the DESeq2 pipeline, and functional overrepresentation was determined using GOseq (68, 69) within R. All transcriptional analyses reported for one isolate were calculated in reference to the other isolate and vice versa. Additionally, visualization of overrepresented pathways were drawn within R.

### Statistical analysis.

Statistical analyses were performed using GraphPad Prism. Two-tailed paired or unpaired Student’s *t* tests were used to compare the means of two samples as stated within this article or one-way analysis of variance (ANOVA) to compare the means of more than two samples. Tukey’s posttest was applied to the *P* value to account for multiple comparisons of the data. *P* values of <0.05 were considered statistically significant.

### Data availability.

Raw data files for these analyses are deposited in BioProject under accession no. PRJNA477447, as previously published by our research group (28). SRA files can be found under accession no. SRS3447362 (NCPF 8973) and SRS3447363 (NCPF 8978).
