# Supplementary figures and images for: Candida auris Phenotypic Heterogeneity Determines Pathogenicity In Vitro
Source: mSphere. 2020 Jun 24;5(3):e00371-20. doi: 10.1128/mSphere.00371-20 (PMC7316489; doi:10.1128/mSphere.00371-20)

**Supplementary Figure 1**


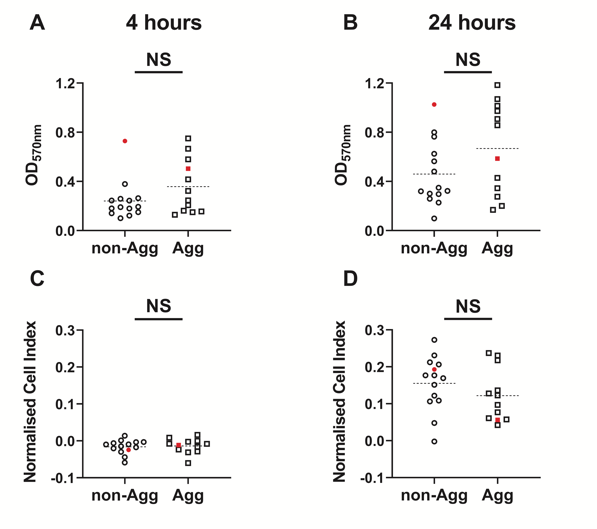

Supplement: FIG S1 [file mSphere.00371-20-sf001.docx]

**Supplementary Figure 2**

***
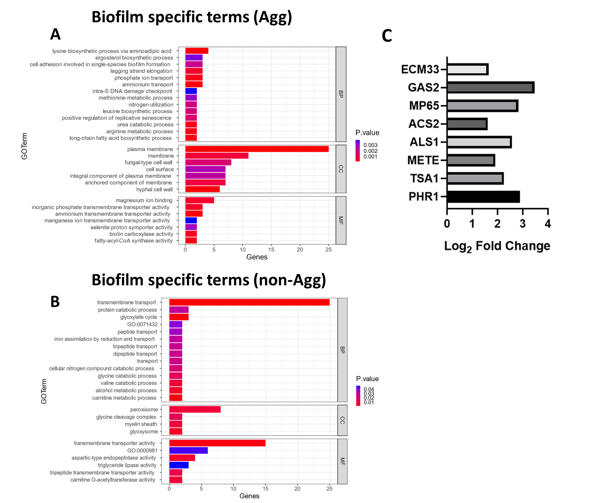
***

Supplement: FIG S2 [file mSphere.00371-20-sf002.docx]

***
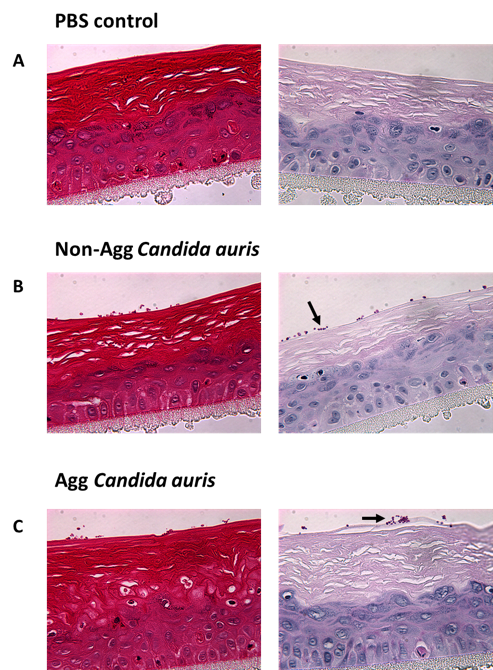
***

Supplement: FIG S3 [file mSphere.00371-20-sf003.docx]
